# Mid- to late Holocene sea-level rise recorded in Hells Bells ^234^U/^238^U ratio and geochemical composition

**DOI:** 10.1038/s41598-023-36777-y

**Published:** 2023-06-20

**Authors:** Nils Schorndorf, Norbert Frank, Simon M. Ritter, Sophie F. Warken, Christian Scholz, Frank Keppler, Denis Scholz, Michael Weber, Jeronimo Aviles Olguin, Wolfgang Stinnesbeck

**Affiliations:** 1 Institute of Earth Sciences, Heidelberg, Germany; 2 Institute of Environmental Physics, Heidelberg, Germany; 3 Heidelberg Center for the Environment (HCE), Heidelberg, Germany; 4 Institute for Geosciences, Mainz, Germany; 5 Museo del Desierto, Saltillo, COAH Mexico; 6 Grupo Espeleologico Ajau, Mérida, YUC Mexico

**Keywords:** Environmental sciences, Geochemistry

## Abstract

Hells Bells are underwater secondary carbonates discovered in sinkholes (cenotes) southeast of Cancun on the north-eastern Yucatán peninsula, Mexico. These authigenic calcite precipitates, reaching a length of up to 4 m, most likely grow in the pelagic redoxcline. Here we report on detailed ^230^Th/U-dating and in-depth geochemical and stable isotope analyses of specimens from cenotes El Zapote, Maravilla and Tortugas. Hells Bells developed since at least ~ 8000 years ago, with active growth until present day. Initial (^234^U/^238^U) activity ratios (δ^234^U_0_) in Hells Bells calcite decreas from 55 to 15‰ as sea level converges toward its present state. The temporal evolution of the geochemistry and isotope composition of Hells Bells calcites thus appears to be closely linked to sea-level rise and reflects changing hydrological conditions (desalinization) of the aquifer. We suggest that decelerated leaching of excess ^234^U from the previously unsaturated bedrock traces Holocene relative sea-level rise. Considering this proxy, the resulting mean sea-level reconstruction contains half as much scatter, i.e. improves by a factor of two, when compared to previously published work for the period between 8 and 4 ky BP.

## Introduction

**Figure 1:**
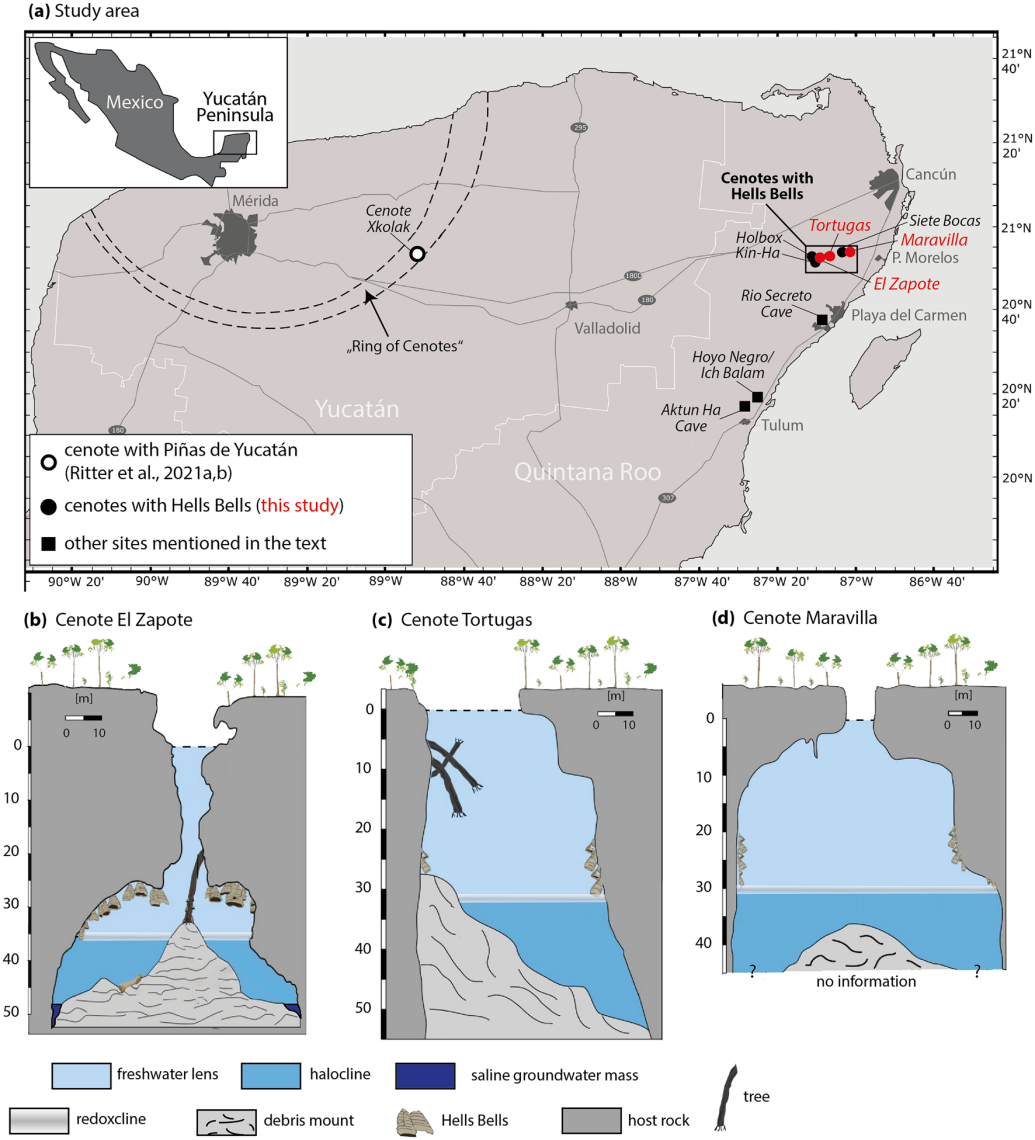
The study area and schematic cross-sections of the investigated cenotes: (**a**) map of the study area with respect to the Yucatán peninsula and Mexico. The map was taken from Ritter et al.^5^ and slightly modified. (**b**–**d**) Cross-section of the cenotes El Zapote, Tortugas and Maravilla showing the host rock and the sedimentary deposits, such as debris, and Hells Bells speleothems. The gray shaded area at the freshwater-halocline interface refers to the redoxcline, where modern carbonate precipitation is suspected according to Ritter et al.^2^ and Stinnesbeck et al.^1^. Note that the water level within the cenotes is roughly equal to mean sea level.

Impressive bell-shaped speleothems hanging from cavern ceilings and walls were recently discovered in ~ 20–40 m water depth in a small cluster of sinkholes (e.g., cenote El Zapote) on the north-eastern Yucatán peninsula (YP) in Mexico (Fig. 1)^1^. These up to 4 m long conically downward expanding structures consist of calcite and grow from the cave ceiling and the surrounding walls in a vertically stratified non-intermixing (meromictic) water body. Local cave divers discovered these unusual speleothems and named them “Hells Bells” because of their bell-like shape and their occurrence in a lightless environment at great water depths close to a sulfidic and turbid halocline, separating the seawater from the overlying freshwater lens.

The first publication reporting about Hells Bells speleothems was provided by Stinnesbeck et al.^1^, who conducted detailed analyses on the morphology and structure of Hells Bells. These initial analyses showed, that Hells Bells speleothems are characterized by laminar fabrics of alternating units of elongated dogtooth spar calcite and microcrystalline spar calcite of which the latter indicate either discontinuous Hells Bells growth and/or intermittent dissolution. The major finding of this pioneering study, however, was that Hells Bells grow underwater as indicated by smaller individuals (few centimeters length) which cover a tree that has fallen into the cenote El Zapote around 3.5 ky before present (BP = 1950 Common Era), when the deep sections of the cenote had already been submerged for thousands of years^1^. Furthermore, they concluded that Hells Bells growth is strictly dependent on the elevation of the halocline, which may offer a wide potential for the use of Hells Bells as a paleo-hydrological archive on the YP.

Ritter et al.^2^ reported detailed hydrogeochemical profiles, chemical and optical analyses of Hells Bells speleothems as well as scanning electron microscope analyses on filtrates of the turbid layer of cenote El Zapote. These authors proposed that the actual growth of Hells Bells is most likely restricted to the 1–2 m thick pelagic redoxcline above a sulfidic halocline in the lowermost portion of the freshwater lens. Based on the results of this comprehensive study, a biologically induced authigenic calcite precipitation within the turbid layer was hypothesized^2^. Recently, Leberecht et al.^3^ showed that microorganisms could metabolically promote authigenic calcite precipitation within the redoxcline, supporting the hypothesis of Ritter et al.^2^. An alternative hypothesis on the formation of Hells Bells speleothems in cenote El Zapote was suggested by López-Martínez et al.^4^, who proposed that Hells Bells represent folia speleothems that form as a result of subaqueous calcite precipitation around CO_2_ bubbles trapped below overhanging walls of the cave^4^. However, this hypothesis is mainly based on the observation of structures at the cave ceiling that were interpreted as bubble trails. Due to the comprehensive work of the aforementioned studies^1–3^, we regard the model proposed by Ritter et al.^2^ as the currently most plausible explanation of Hells Bells growth.

By comparing the hydrology of cenotes with and without Hells Bells, Ritter et al.^2^ postulated certain prerequisites for the formation of Hells Bells, such as stagnancy of the water body or sufficient input of organic material to create anoxic conditions within the halocline, among others. Considering these fundamental requirements, it was predicted that suitable conditions for the formation of Hells Bells were likely to be found in other deeply stratified cenotes on the YP. This assumption was confirmed in 2020, when a team of cave divers and scientists discovered brownish calcitic coatings forming pinecone-like structures at overhangs below ~ 52 m water depth in cenote Xkolac on the northwestern YP (Fig. 1a)^5^, reinforcing the hypothesis of Ritter et al.^2^. Because of their pinecone-like appearance these formations were termed as “Piñas de Yucatán” and it was suggested that both, Hells Bells and Piñas de Yucatán, might represent a novel sub-type of underwater speleothems, which are formed by microbially promoted calcite precipitation in pelagic redoxclines and could therefore be termed “redoxithems”^5^.

So far, only two small Hells Bells specimens have been dated by means of mass spectrometric uranium–thorium dating method (^230^Th/U) yielding ages between 5200 and 300 years BP^1,4^, and their timing and growth rate have thus remained uncertain. Therefore, we conducted a systematic study using ^230^Th/U-dating on several Hells Bells from the cenotes El Zapote, Maravilla and Tortugas on the north-eastern YP. This includes samples collected from the cave walls at different water depths (e.g. “TL4” from the turbid layer in cenote El Zapote at 35.8 m water depth or “MIII” from cenote Maravilla at 29.4 m water depth), samples collected from the bottom of cenote El Zapote (e.g. “Big Bell” or “ZPT-7”) and samples grown on a drowned tree trunk (“Tree Bells”) between 32.7 and 37.3 m water depth. In addition, we investigated the geochemical composition of these speleothems (including major and trace elements, as well as stable carbon and oxygen isotopes). Our findings reveal a systematic temporal trend in Sr/Ca ratios and initial (^234^U/^238^U) activity ratios across all three cenotes. This trend appears to be strongly associated with the final phase of relative sea-level rise during the mid to late Holocene. Multiple studies have previously suggested a link between relative sea level and seawater initial (^234^U/^238^U) activity ratios using corals^6–9^, whereas our study investigates local variations within a freshwater lens overlying seawater.

(^234^U/^238^U) activity ratios are reported here in delta notation (δ^234^U values), representing the deviation of (^234^U/^238^U) from secular equilibrium (δ^234^U = (^234^U/^238^U)−1). The initial value of δ^234^U (δ^234^U_0_) can be calculated using the ^230^Th/U age t:

1 $$\delta^{234} U_{0} = { }\delta^{234} U_{m} {*}e^{{\lambda_{234} *t}}$$

In groundwater, ^238^U and ^234^U are commonly found to be in radioactive disequilibrium (e.g.^10,11^). The δ^234^U_0_ value is influenced by the alpha-recoil process, host rock dissolution, and redox-behavior of Uranium^12–15^. In a recent study of δ^234^U values in subaqueous carbonate deposits from Devils Hole, southwest Nevada (USA), it was proposed that even under dry conditions (e.g., during periods of a low water table), alpha-recoil causes excess ^234^U to accumulate in damaged crystal lattice sites and/or on fractured surfaces of the bedrock and sediments, which may then be ‘captured’ as the water table returns^16^. These observations suggest that Hells Bells δ^234^U values may be linked to past water levels in cenotes of the YP.

## Results

### Hydrogeochemistry of cenote El Zapote

Uranium concentrations are nearly constant at ~ 3 µg L^−1^ in the upper 35 m of the oxygenated freshwater lens and instantly drop by an order of magnitude as soon as the water becomes anoxic (see Supplementary Fig. S1). δ^234^U values of 16 ± 1‰ are nearly constant throughout the freshwater lens and increase within the halocline to 60 ± 3‰ at 45 m water depth. Uranium concentrations are anticorrelated with δ^234^U values (r_U/δ234U_ =  − 0.68, p < 0.05).

### ^230^Th/U-ages and δ^234^U_0_ values

**Figure 2:**
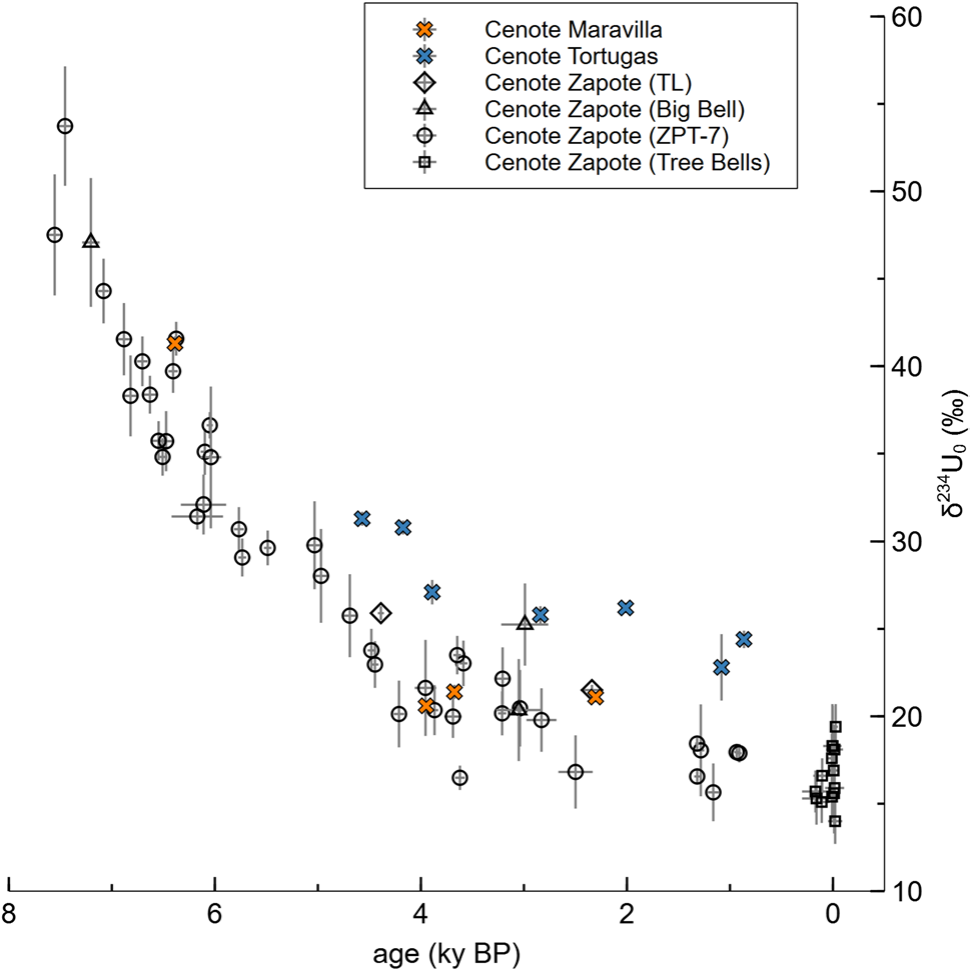
Uranium isotopes of Hells Bells samples: δ^234^U_0_ values versus age of Hells Bells samples dated to < 8 ky. Uncertainties are given as 2σ margins.

^230^Th/U-dating of the studied Hells Bells yielded ages from 95.81 ± 0.52 ky BP to modern (see Table S1). Initial δ^234^U_0_ values vary from 15.1 ± 1.2 to 62.6 ± 4.6‰, with the highest values observed in the early Holocene and systematically decreasing values for the mid- to late Holocene (see Table S1). In fact, δ^234^U_0_ values steadily decline in all studied Bells from three different cenotes from 55‰ to around 20‰ between ~ 8 and 4 ky BP, followed by a minor decrease to values of ~ 15‰ to present (Fig. 2). Overall, δ^234^U_0_ values from various Hells Bells and cenotes thus reveal an identical temporal variability, which must reflect the isotopic composition of the water, in which those carbonates have formed, since there is no fractionation during the incorporation of uranium into carbonates^17^.

**Figure 3:**
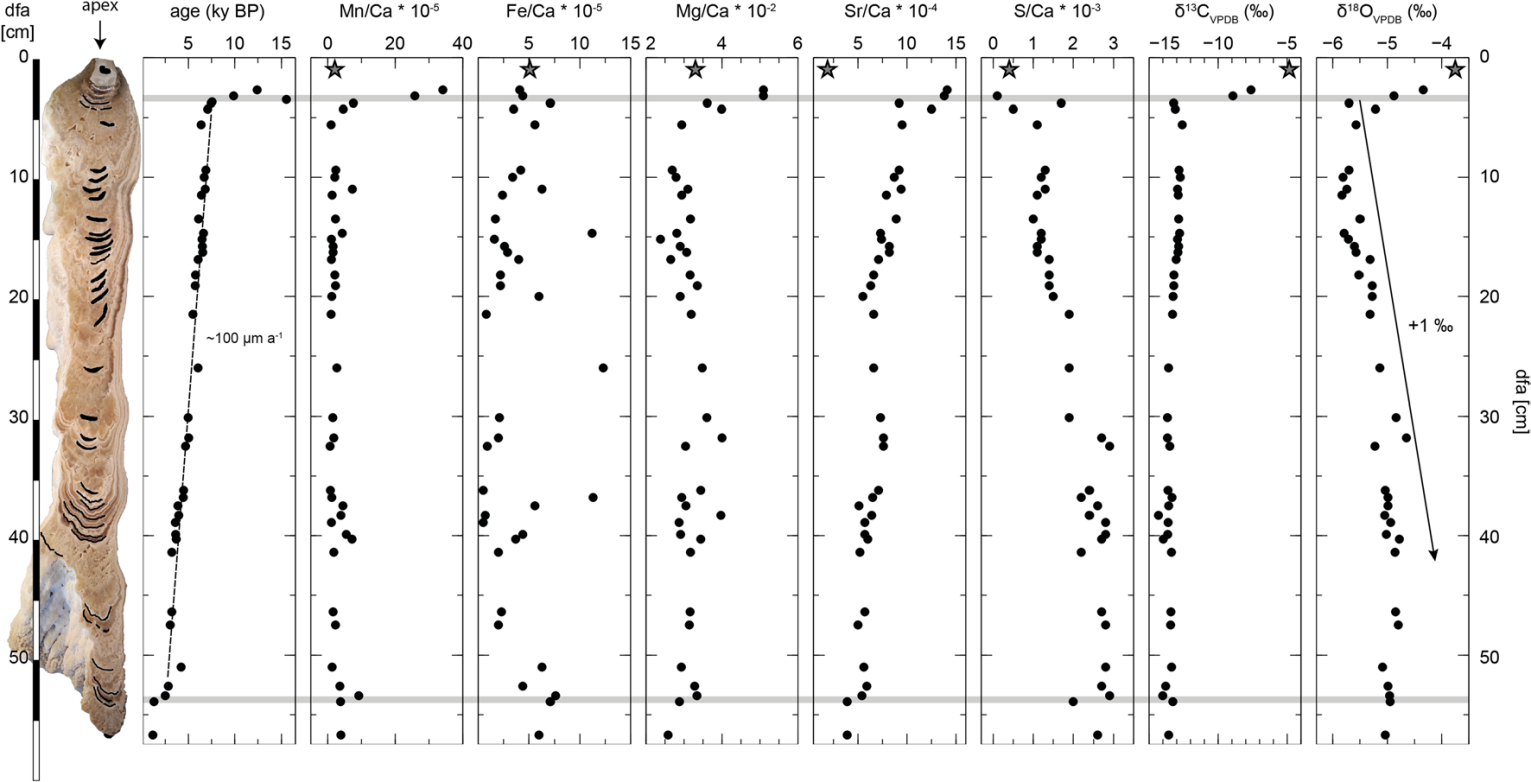
Ages and geochemistry of ZPT-7 along the growth axis: Apical root ends up with carbonate host rock (star symbol). Sample spots are indicated by blackened areas in the image on the left. The major growth discontinuities in the uppermost part as well as in the lower part of the bell are highlighted by gray bars. Note the increasing trends in δ^18^O values and molar S/Ca ratios as well as the decreasing trends of Sr/Ca ratios and δ^13^C values. The ages and geochemical results are presented in Supplementary Table S1 and 2. dfa, distance from the apex.

As expected, the root of Big Bell reveals the oldest growth phase dating back to 95.81 ± 0.52 ky BP (see Supplementary Fig. S2). The growth of ZPT-7 starts on top of the marine limestone slab (host rock) (Fig. 3; Supplementary Figs. S2 and S3). The determined ages are mostly in stratigraphic order. Layers within ~ 3.5 cm distance from the apex (dfa), yielded ages between 15.5 and 10 ky BP (see Fig. 3, Supplementary Figs. S2 and S3). One of these samples was taken immediately adjacent to a growth interruption, which is macroscopically identified as a black rim conformed by Fe-sulfide (pyrite) (see Supplementary Fig. S2). This sample yielded a significantly older age (15.51 ± 0.21 ky BP) than the two samples above, thus representing a major stratigraphic inversion. A similar age (15.02 ± 0.13 ky BP) was obtained from a sample of the root of Big Bell, which was also sampled near a thin layer of Fe-sulfide (see Supplementary Fig. S2). From 7.7 to 2.5 ky BP, i.e., from 3.7 to 53.5 cm dfa, the ages of ZPT-7 suggest more regular growth with an average growth rate of about 100 µm year^−1^ (Fig. 3, Supplementary Fig. S3). At 2.5 ky (53.5 cm dfa), a growth interruption is evident with a duration of c. 1000 years (Fig. 3, Supplementary Fig. S3). The tip of ZPT-7 at 56.7 cm dfa is dated to an age of 1.284 ± 0.083 ky BP, which possibly corresponds to the timing of bell downfall. ^230^Th/U-dating of the antapical ends of Tree Bells collected from water depths between 32.7 and 37.3 m yielded ages between 0.17 ± 0.13 and − 0.026 ± 0.021 ky BP (Supplementary Fig. S2). Moderate uranium concentrations together with high detrital ^232^Th and a low (^230^Th/^232^Th) activity ratio result in relatively large age uncertainties of these samples, which, however, are most likely modern.

### Geochemistry of Hells Bells

**Figure 4:**
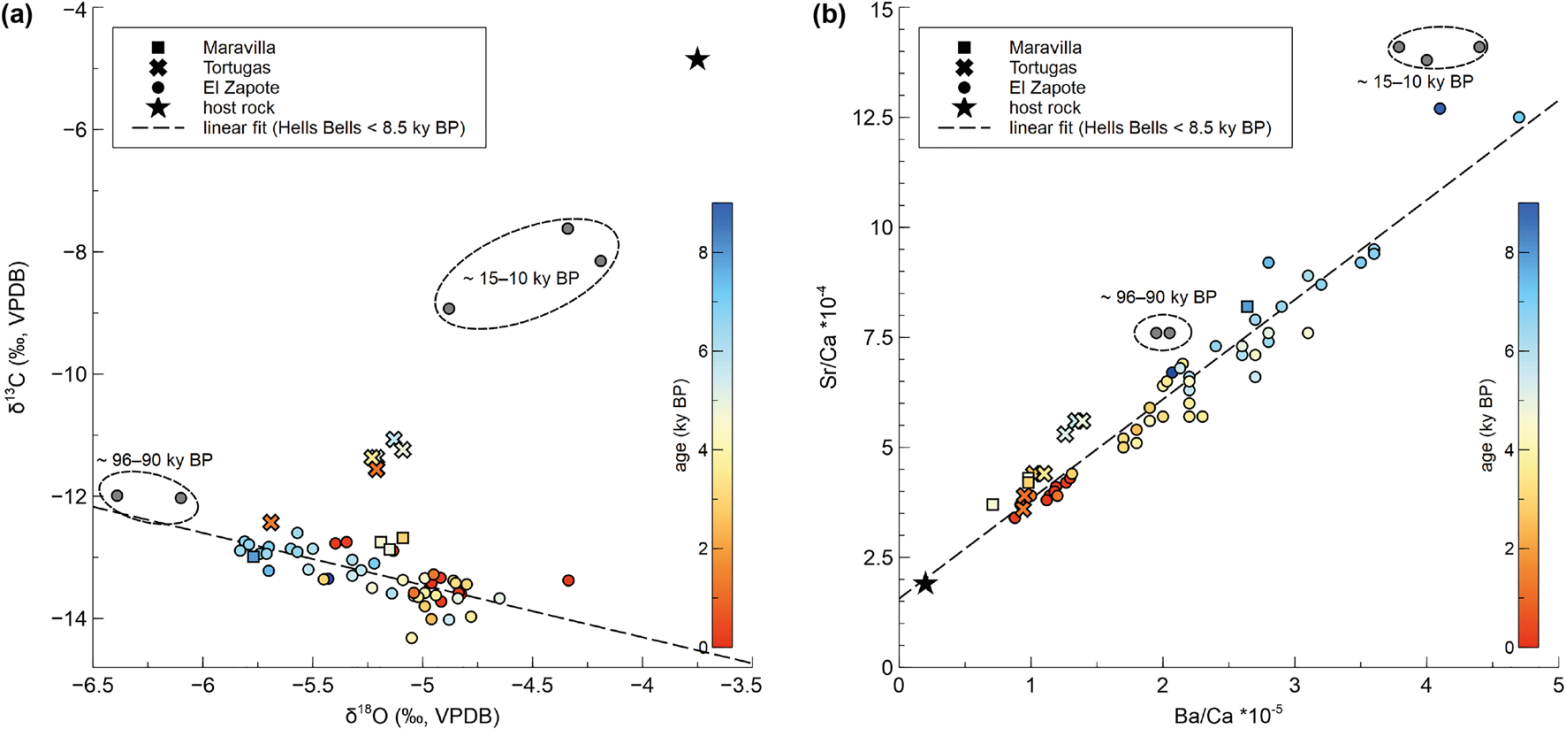
Geochemistry of all analyzed Hells Bells samples: (**a**) stable carbon isotope values (δ^13^C) plotted against stable oxygen isotope values (δ^18^O) of all analyzed Hells Bells samples, and the host rock sample from the root of ZPT-7 (El Zapote cenote). (**b**) Sr/Ca against Ba/Ca molar ratios of all analyzed Hells Bells samples and the host rock sample. Sr and Ba contents of Hells Bells calcite are linearly correlated and indicate that the initial Sr/Ba ratio of the host rock is preserved in Hells Bells carbonates. The different symbols represent the different cenotes. Samples < 8.5 ky BP are color-coded depending on their respective age. The geochemical results can be found as Supplementary Table S2.

The host rock carbonate shows stable carbon and oxygen isotope values (δ^13^C and δ^18^O values) of − 4.9‰ and − 3.8‰, respectively. The δ^13^C values of the Hells Bells samples range from − 14.3 to − 7.6‰, with the majority of the samples ranging from − 14.3 to − 11.1‰. The stable oxygen isotopes (δ^18^O values) of Hells Bells range from − 6.4 to − 4.2‰. Hence, the δ^13^C and δ^18^O values of Hells Bells are different from those of the host rock. Most samples form a distinct cluster in the stable isotope plot (Fig. 4a), which corresponds to the mid- to late Holocene samples. Here, δ^13^C values correlate inversely with δ^18^O values (r_δ13/ δ18O_ =  − 0.7, p < 0.05). There are only a few samples from cenote Tortugas that deviate from this trend, as they exhibit slightly less negative δ^13^C values (− 11.4‰ on average) than the other samples (− 13.1‰ on average). The two oldest samples (~ 96 and 90 ky BP) also plot closely to this cluster and only slightly differ from the rest by showing the most negative δ^18^O values (− 6.4‰ and − 6.1‰). The trend in the isotope correlation is also visible through time, with decreasing δ^13^C values and increasing δ^18^O values towards younger ages (Figs. 3 and 4). The three samples with ages between ~ 15 and 10 ky BP, however, deviate clearly from this trend by showing the most positive δ^13^C values (− 8.9‰ to − 7.6‰).

Hells Bells carbonates show strongly correlated Sr/Ca and Ba/Ca ratios (r_Sr/Ba_ = 0.97; p < 0.05), and the linear fit intersects with the Sr/Ca and Ba/Ca ratio of the host rock (Fig. 4b). The ratios of these samples show a decreasing trend with increasing dfa and decreasing age, respectively (Fig. 4b). As for the stable isotopes, the Sr/Ca and Ba/Ca ratio of the older samples (96–90 ky BP and 15–10 ky BP) show slightly different values than the general trend.

Dark, brown-colored layers identified on the polished half of ZPT-7 correspond to elevated Mn/Ca and Fe/Ca values (Fig. 3) and show a large scatter. The molar ratio of Mg/Ca (2.4–5.1 × 10^–2^) appears rather constant through time (Fig. 3). In contrast to the metal/calcium ratios, the multivalent non-metal sulfur (S) reveals an opposing trend with an increasing S/Ca ratio of ZPT-7 from ~ 0.1 to 2.7 × 10^–3^ with decreasing age (Fig. 3). These geochemical trends are thus evident throughout all studied Hells Bells of cenote El Zapote as well as in the nearby cenotes Maravilla and Tortugas (Fig. 4).

## Discussion

### ^230^Th/U-chronology and Hells Bells growth

Radiometric dating traces the age of carbonate precipitation, and in accordance with relative sea-level elevation, a Middle to Late Holocene phase of Hells Bells growth is identified. During most of the last 8.5 ky, ^230^Th/U-dating provides clear evidence for partly continuous growth of Hells Bells, for example, from 7.5 to 2.5 ky BP in ZPT-7 (Fig. 3). Furthermore, the roots of Big Bell and ZPT-7 revealed some minor growth during the last interglacial and possibly during the Pleistocene/Holocene transition (c. 15–10 ky BP). However, these few older ages are unlikely reliable for its relation to the water table and the massive age inversions. Considering reconstructions of relative sea level (e.g.,^18,19^), these ages would indicate that Hells Bells grew at or above the water table, contradicting submerged growth of these redoxithems^2^. One possible explanation is subaerial weathering of even older Hells Bells i.e., dissolution or secondary carbonate overprinting from meteoric water, during the sea-level low-stand of the last glacial, leading to false or mixed ages^20^. As an alternative, these first growth layers may not even represent Hells Bells, but instead growth that is similar to carbonate encrustations, such as phreatic overgrowths on speleothems (POS), which can precipitate at the water level in a brackish environment (e.g.,^21^). This could also explain the slight differences in geochemical composition from the other samples (see Fig. 4). Further, we do not know whether the Fe-sulfide layers within the roots of Big Bell and ZPT-7—or the processes that led to their formation—may have had an influence on the geochemistry of the adjacent calcite layers and thus the ^230^Th/U ages. Consequently, these earlier (Pleistocene) and very minor growth layers bear a large risk of weathering influences and overprinting, leading to un-reliable ages and geochemical composition. We therefore focus on the discussion of the significant findings of the mid- to late Holocene samples in the following.

The ^230^Th/U-dating results of ZPT-7 show that Hells Bells calcite can be dated at century scale resolution (Fig. 3 and Table S1). Minor age inversions identified throughout the specimen are likely due to the complex internal cauliflower structure of Hells Bells, which makes a continuous track of the growth axis during sample collection difficult. The here observed mid- to late Holocene timing of Hells Bells growth confirms previous punctuated observations^1,4^. Between 7.5 and 2.5 ky BP (3.7–53.4 cm dfa), ^230^Th/U-ages of ZPT-7 show an average growth rate of about 100 µm year^−1^ (Fig. 3). This growth rate is in the same order of magnitude as the one estimated from ^230^Th/U-dating by Stinnesbeck et al.^1^ and two orders of magnitude higher than other types of subaqueous speleothems, such as mammillary calcite or folia^22–24^. The hand-sized Hells Bells speleothem TL4 from El Zapote and the ones from Maravilla and Tortugas show lower growth rates (~ 4–18 µm year^−1^) as compared to ZPT-7 (~ 100 µm year^−1^). This rather unsteady growth is supported by the strong lamination of these samples (see Supplementary Fig. S4). These differences in growth rate could be explained on the basis that the bells were probably hanging at water depths where fluctuations of the halocline caused them to coincide with the position of the redoxcline to a different extent. In cenote El Zapote, for example, Hells Bells appear to grow faster in the central part of the 10 m zone of Hells Bells appearance (~ 28–38 m water depth), which is further visually evident by the size distribution of Hells Bells with water depth^1,25^. There may be other minor contributing factors or processes (e.g. topographical variations of the rock from which Hells Bells are growing) that could have an impact on the growth rates of Hells Bells, but this is beyond the scope of this study and requires further investigation. The ages of 3.05 ± 0.22 ky BP and 1.162 ± 0.054 ky BP, determined for the lowermost parts of Big Bell and ZPT-7, respectively, may refer to the times when these specimens broke off the cave ceiling and fell on the cave floor, where they stopped growing. Whether these break-offs were gravitationally triggered by the weight of the Bells, or even by a devastating event (e.g., earthquake), remains speculative.

Verification of recent growth of Hells Bells is challenging considering the low growth rates and the partly high concentrations of ^232^Th (up to 6 ng g^−1^) in Tree Bell samples. Nevertheless, the 2–3 mm thick samples collected from water depths between 32.7 and 37.3 m yield very young ages of a few decades to centuries (Supplementary Fig. S2). Thus, we suggest the growth of Hells Bells to be presently active and that the elevation of the halocline, and thus, the zone of Hells Bells growth, varied on the scale of several meters within this period (few decades to centuries).

Overall, the results of the ^230^Th/U-dating on different Hells Bells specimens from different cenotes on the YP thus reveal at least semi-continuous growth of Hells Bells since about 8.5 ky BP until present.

### Stable carbon and oxygen isotopes

**Figure 5:**
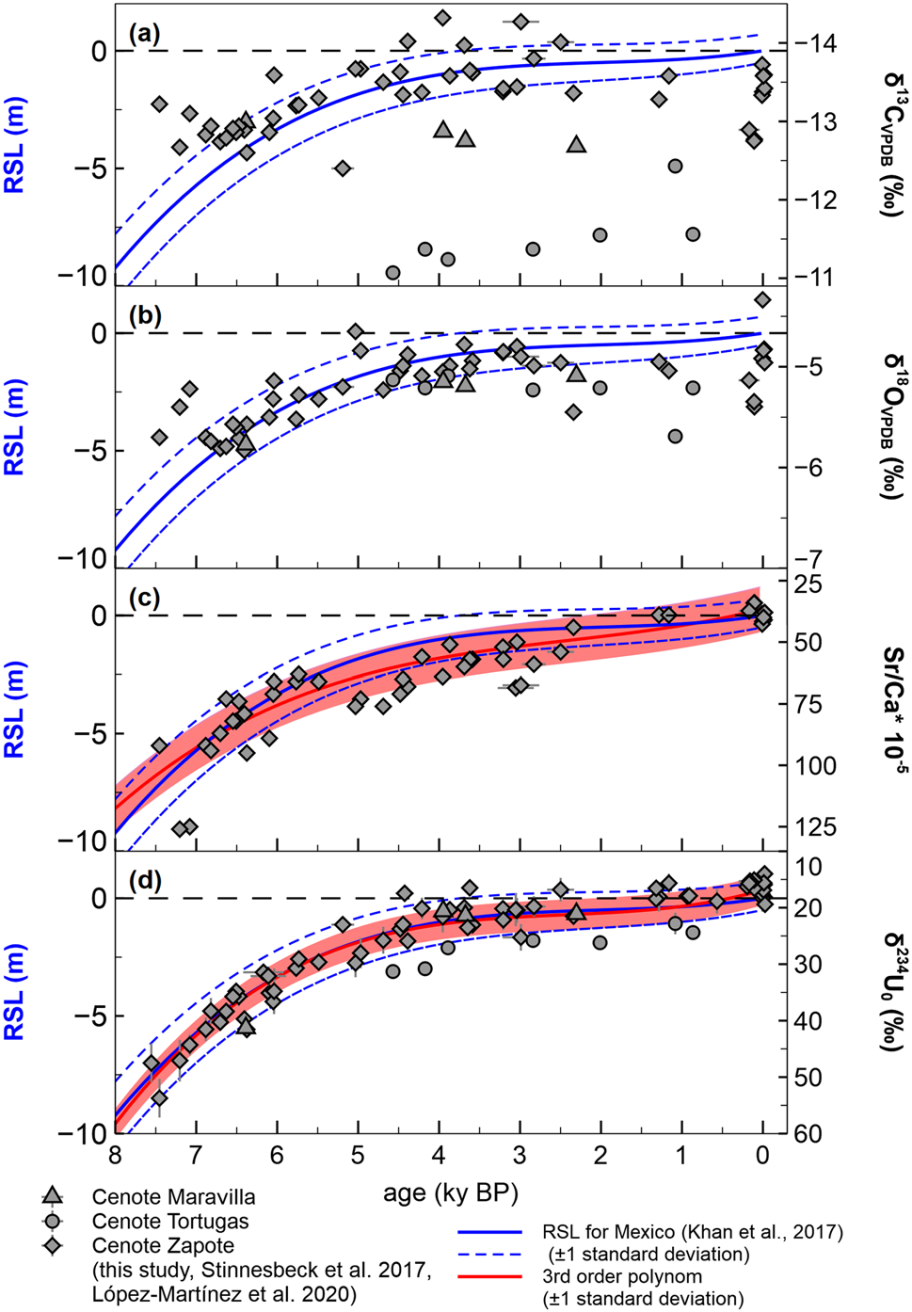
Hells Bells geochemistry and relative sea level: (**a**–**d**) geochemistry (δ^13^C-, δ^18^O-, δ^234^U_0_-values and Sr/Ca ratio) of Hells Bells from cenotes El Zapote, Maravilla and Tortugas during the past ~ 8 ky. The very few Hells Bells data available so far from Stinnesbeck et al.^1^ and López-Martínez et al.^4^ are shown as well. Uncertainties are given as 2σ margins. The red curves are a 3rd order polynomial fit through Hells Bells Sr/Ca ratios and δ^234^U_0_ values, respectively. The blue curve is a 3rd order polynomial fit through relative sea-level data for Mexico at a 1000-year-interval during the past 8 ky estimated by the empirical hierarchical model of Khan et al.^14^.

Most Hells Bells samples reveal δ^13^C values ranging from − 14‰ in cenote El Zapote, to − 11‰ in cenote Tortugas (Figs. 4a and 5a). The dissolved CO_2_ in the redoxcline is fueled by organic matter decomposition in the anoxic saline groundwater mass and host rock dissolution buffering the acid produced in microbial organic matter decay via sulphate reduction^2^. Consequently, changes in δ^13^C values could reflect a change in vegetation type (C_3_/C_4_ plants), a change in vegetation density (pCO_2_ of the soil), a change in carbon source (organic matter vs. host rock), or a combination of all of them. δ^18^O values in Hells Bells speleothems slightly increase from − 5.8 to − 5.0‰ between ~ 7–3 ky BP (Fig. 5b). Here, the oxygen isotopes appear to increase during the final rise in relative sea level. However, from ~ 3000 years to present, δ^18^O values decrease again, contrasting an already stabilized sea level. Additionally, δ^18^O values of the most recent Hells Bells samples (< 0.17 ± 0.13 ky BP) from cenote El Zapote also exhibit variations of similar amplitude as the entire time series of ± 1‰ (Fig. 5b). Therefore, sea level cannot be the cause of groundwater and, thus, Hells Bells δ^18^O changes. Consequently, other influences must be considered in order to understand the bells δ^18^O variations.

Since it was proposed that Hells Bells are growing within a 1–2 m thick pelagic redoxcline in the lowermost portion of the freshwater lens^2^, the stable oxygen isotope record measured in Hells Bells calcite mainly depends on the isotopic composition of the meteoric groundwater (freshwater lens). The freshwater’s isotopy is dependent on (1) the isotopic composition of precipitation, since groundwater on the YP is a long-term integrator of precipitation and infiltration^26^, and (2) to a lesser extent on molecular viscous diffusion between the saline groundwater (δ^18^O =  − 0.3 to 1.3‰^26^) and the freshwater lens (δ^18^O =  − 8.8 to 6.8‰^26^). A three-year monitoring of drip- and groundwater in the Rio Secreto Cave near Playa del Carmen about 30 km south of our study area (e.g., El Zapote, Fig. 1a), showed that the δ^18^O values of groundwater are consistent with the annual amount-weighted δ^18^O value of rainfall (δ^18^O =  − 4.7 ± 0.1‰), while its temporal isotopic stability suggests that it integrates several years of rainfall^27^. In the tropical Atlantic region, δ^18^O values of precipitation are generally linked to summer rainfall amount, a relationship which is based on the type and source of wet season (convective) versus drier season (orographic) rainfall^28–30^. Convective rainfall during the wet summer season associated with frequently occurring tropical storms and hurricanes shows characteristic depleted isotopic values^30^. A study in the northwestern part of the YP showed that the depleted isotopic composition associated with a single hurricane event can disturb the baseline δ^18^O value of groundwater for a few years^31^. The slight increase in Hells Bells’ δ^18^O values between ~ 7 and 3 ky BP (Fig. 5b) agrees well with stalagmite records from Guatemala^32^ and Mexico^33^, indicating higher precipitation amounts and/or convective intensity during the mid-Holocene, followed by a minor drying trend towards the Late Holocene. Based on these findings, changes in δ^18^O values of Hells Bells calcite most likely represent long-term changes of local precipitation amount and/or convective activity.

### Hells Bells geochemistry, sea level and freshwater lens thickness

Here, we presume Hells Bells growth depends on the position of the halocline according to the model of Ritter et al.^2^ which infers an interplay between relative sea-level elevation and the thickness of the overlying freshwater lens (hydrostatic pressure). Consequently, it can be assumed that changes in either of the two drivers are reflected in the geochemistry of Hells Bells calcite. The Sr/Ca ratio of the freshwater lens within the Hells Bells cenotes is mainly controlled by host rock dissolution and molecular viscous diffusion from the underlying saline groundwater.

In a study from Aktun Ha Cave, about 75 km south of the Hells Bells cenotes (Fig. 1a), changes in the benthic microfossil assemblages indicated a gradual decrease in freshwater salinity over the past ~ 7 ky^34^. The authors of this study suggested that the decrease in freshwater salinity might be related to turbulent mixing at the halocline due to increased flow in the freshwater lens^34^. Instrumental monitoring within cave systems and cenotes on the north-eastern YP has shown that heavy rainfall events (i.e., hurricanes) can lead to an increased groundwater salinity for several weeks to months after the events^35–37^. Similar to the study of van Hengstum et al.^34^, Sr/Ca and Cl/Ca ratios of calcite raft deposits in cenotes Ich Balam and Hoyo Negro (Fig. 1a), show a continuous decline in the salinity of the freshwater lens over the past ~ 7 ky^38^. Calcite rafts form in CaCO_3_ saturated water at the air–water interface through CO_2_ degassing and evaporation. They form conically shaped piles (raft cones;^39^) as they sink and accumulate on the cave bottom, thereby providing records of the upper freshwater lens^40^. Kovacs et al.^38^ also suggest that the decline in aquifer salinity during the last ~ 7 ky might be related to reduced freshwater flow in the aquifer, reflecting a change in hydrology (drying trend). However, both studies also noted that karst properties and decelerating Holocene sea-level rise are likely contributing factors^34,38^.

Regarding the Hells Bells cenotes, turbulent mixing at the halocline is unlikely, since the hydrological system is highly stagnant and purely laminar. This stagnancy is shown very well in the density gradient and Sr/Ca values of the water column within cenote El Zapote (see Supplementary Fig. S1). Cenotes with Hells Bells all show stagnant water bodies with a low degree of advection, thick haloclines (~ 10–19 m) and diffusion-dominated mass transport^25^. Moreover, frequent turbulent mixing events from intense precipitation would eliminate any calcite supersaturation within the redoxcline and thereby inhibit the formation of Hells Bells. Thus, mixing between the different water masses within the Hells Bells cenotes is only controlled by molecular diffusive processes.

However, the large spatial extent of systematic geochemical changes of the freshwater lens and calcite chemistry is well expressed by Hells Bells samples from the cenotes El Zapote, Maravilla and Tortugas, which, like the calcite rafts from cenotes Ich Balam and Hoyo Negro^38^, show consistently decreasing Sr/Ca ratios and thus a decrease in the salinity of the freshwater lens during the last ~ 8 ky (Fig. 5c). Reconstructions of mid- to late Holocene relative sea-level rise in Mexico show that sea level increased by roughly 8–10 m during the last ~ 8 ky (Fig. 5)^18^. The deceleration of sea-level rise matches with the decreasing pattern of Hells Bells Sr/Ca ratios with an average deviation of 8% from the sea-level fit (Fig. 5c).

Over the past ~ 8 ky, δ^234^U_0_ values decrease continuously from values around 55–60‰ to values between 15 and 20‰, as sea level converges toward its present state (Fig. 5d). This pattern of δ^234^U_0_ values matches the progression of middle to late Holocene relative sea-level rise on the YP even better than that of Sr/Ca ratios, presenting an average deviation of only 6% from the relative sea-level fit (Fig. 5d). δ^234^U_0_ values of Hells Bells calcite reflect the (^234^U/^238^U) activity ratios of the paleo water in which they were formed. Several mechanisms may be involved in controlling δ^234^U values of the aquifer such as the alpha-recoil process, host rock dissolution, and redox-behavior of Uranium^12–15^. Hence, the origin and variability of freshwater δ^234^U values as well as the U concentration is difficult to assess. Regarding Hells Bells speleothems, isotopic variations are very systematic and occur over large spatial scales. Isotopically enriched U is supplied through diffusion from the underlying saline groundwater body, even if the concentration of uranium is reduced here due to anoxic conditions in which U behaves particle reactive. In contrast, today’s overlying freshwater lens seems rather homogeneous and is close to secular equilibrium (~ 16‰; see Supplementary Fig. S1). In a recent study from Devils Hole 2 cave (Nevada, USA), δ^234^U_0_ values of subaqueous calcite were interpreted as a proxy for water–rock interactions in the regional aquifer^16^. Wendt et al.^16^ propose that changes in the elevation of the water table are responsible for changes in the amount of leached excess ^234^U from the bedrock, and that variations in (^234^U/^238^U) activity ratios coincide with interglacial-glacial cycles. Although the hydrology of the Yucatán Karst Aquifer is distinctly different from that of the Devils Hole in southwest Nevada, they both are subject to recurrent changes in water level elevation on interglacial–glacial timescales. In Nevada, water table fluctuations are driven by variations in recharge amount to the local groundwater flow system^41^, whereas on the YP, they are associated with glacio-eustatic changes in sea level^42^. Similarly to the findings of Wendt et al.^16^, we advocate that the inundation of previously unsaturated bedrock causes a concomitant change in δ^234^U values of the groundwater with relative sea level. Upward diffusion of U into the freshwater layer contradicts the concentration gradient of U (see Supplementary Fig. S1), but could provide slow isotope exchange. Hence, alternatively a growing volume of the freshwater lens would not only account for a subsequent decrease of excess ^234^U but also decreasing Sr/Ca ratios. However, this interpretation contradicts the decreasing trend in precipitation as indicated by the Hells Bells’ δ^18^O values. In spite of an alternative explanation, we suggest that the evolution of δ^234^U values can be used as a regional proxy for relative sea-level changes.

## Conclusions

Data obtained from ^230^Th/U-dating, geochemical and stable isotope analyses of several Hells Bells specimens from the cenotes El Zapote, Maravilla and Tortugas on the north-eastern Yucatán peninsula, show that geochemical records of subaqueously grown Hells Bells speleothems provide a proxy for paleo-hydrological conditions of the local aquifer. ^230^Th/U-dating of small Hells Bells knobs growing on a drowned tree trunk suggest that growth reaches to modern times and is an ongoing process. Geochemical (Sr/Ca ratios) and isotopic trends of δ^234^U_0_ values of Hells Bells calcites over the past ~ 8 ky follow the gradual increase and stabilization of relative sea level. We suggest that this stabilization in sea level towards the late Holocene accounts for a desalinization of the freshwater lens as indicated by decreasing Sr/Ca ratios.

This is the first study to show that δ^234^U_0_ values of Hells Bells coincide with the final Holocene relative sea-level rise in the Caribbean. Along with the deceleration of Holocene relative sea-level rise towards its present state, the contribution of accumulated excess ^234^U to the aquifer decreased equally, thus reflecting a unique empirical relationship. δ^234^U values of Hells Bells calcites may thus provide a valuable proxy for the reconstruction of relative sea level in the Caribbean by reducing the uncertainty of the sea-level curve itself.

## Methods

### Hells Bells samples

The three studied cenotes (El Zapote, Tortugas and Maravilla) are located southwest of Cancún, in the Mexican federal state of Quintana Roo (Fig. 1a). Cenote El Zapote (20°51′27.78″ N 87°07′35.93″ W) is water-filled and connected to the surface by a 28 m deep vertical shaft (Fig. 1b). The freshwater lens and the saline groundwater mass are separated by a thick halocline reaching from 36.7 to 51.7 m water depth (Fig. 1b)^25^. At 28 m depth below the water level, the cave walls diverge almost horizontally and form a 60 to > 100 m wide cavern, reaching to a depth of 54 m^1^. A 20 m high debris mound in the center of the cave is built up by limestone blocks and smaller debris, large stems of jungle trees and other vegetation falling in from the surface, as well as abundant organic matter^1^. Hells Bells hanging from the cavern ceiling and walls and reaching lengths of up to > 4 m appear in water depths of 28–38 m (Fig. 1b)^1^. Cenote Tortugas (20°51′11.7″ N 87°06′30.1″ W) is located 24 km west of Puerto Morelos coast and about 2 km east of cenote El Zapote. The cenote shows a large circular opening of about 25 m diameter and is slightly asymmetric in cross-section (Fig. 1c). A debris mound lies against the wall of one side of the cenote and dips towards the opposite side from 25 to around 60 m water depth. Here, Hells Bells appear in water depths from 25 to 35 m^1^. Cenote Maravilla is located about 16 km west of the coast of Puerto Morelos (20°52′18.9″ N 87°01′24.5″ W). The cenote is bottle-shaped in cross section with a central debris mound (Fig. 1d). Hells Bells appear in water depths from ~ 19 to at least 32 m. The selected Hells Bells are composed of horizontally laminated calcite layers, with the lowermost (youngest) parts of the Bells frequently ending into mm to cm sized, elongated dog-tooth calcite crystals^1,2^.

Here we study three different types of Hells Bells samples from cenote El Zapote. (1) The root and bottom of a 1.8 m long Hells Bells specimen termed Big Bell. This specimen was recovered from the cenote floor in 2017 and is presently displayed at the visitor center of El Zapote Eco Park. A slice of the uppermost root was cut with a diamond saw and was polished subsequently. Samples were drilled perpendicularly to the presumed growth axis with a Dremel tool using a diamond coated stainless-steel drill. Samples from the bottom of Big Bell were homogenized by grinding in an agate mortar. (2) An elongated ~ 60 cm long Hells Bells specimen called ZPT-7 was also collected from the floor of cenote El Zapote^1^. It was vertically cut in half, polished, and samples were drilled along the presumed growth axes and along visual growth layers of about 0.5–1 mm thickness. (3) Some smaller Hells Bells of 5–8 cm size (Tree Bells) grown on a tree trunk were collected in June 2017 from seven water depth levels between 31.3 and 37.3 m. Their geochemical composition (trace elements and stable isotopes) was previously published by Ritter et al.^2^. To obtain the youngest parts of individual Tree Bells, the samples were microscopically studied, and only 2–3 mm thick samples with apparently fresh, well-accentuated crystal tips were chosen for further analyses. The sampling of the small Tree Bell fragments differs from the others since the sample material for ^230^Th/U-dating was not taken as aliquots from a homogeneous powder used for geochemical analysis. Here, sample material for dating was taken close to the areas where sample material for geochemical analysis was collected. In 2018, additional small Hells Bells were collected in cenote El Zapote (TL4), but also from the nearby cenotes Maravilla (MIII) and Tortugas (T7 and T12). They were vertically cut, polished and samples for geochemical analyses were drilled. Aliquots of all samples were taken for the subsequent geochemical analyses. In 2020, six additional Hells Bells were collected from the floor of cenote El Zapote. Samples for ^230^Th/U-dating were drilled from the uppermost root and bottom of these specimens.

### Water samples

In addition to carbonate samples, water at cenote El Zapote was sampled in 2018. Water samples between 0 and 36 m were retrieved using a 0.5 m high Polyethylene FreeFlow bottle (HYDRO-BIOS, Kiel, Germany), and samples between 36 and 52 m were collected by technical divers by drawing up the water of the desired sampling depth into sterile PE-luer-lock syringes (140 mL) with an attached three-way valve^25^.

### ^230^Th/U-dating

In total, 80 Hells Bells samples were analyzed by high-precision ^230^Th/U dating using a multi-collector inductively coupled plasma source mass spectrometer (MC-ICPMS, Thermo Fisher Neptune^plus^) at the Universities of Heidelberg and Mainz. The applied sample treatment, mass spectrometry and data treatment are detailed in previously published work^43–47^. Solid Tree Bell samples were pre-cleaned through a weak acid leach and dried prior to dissolution in 7 N HNO_3_. The chemical preparation consisted of a U and Th purification using UTEVA (HD) or AG1-X8 (Mainz) resin through manual chemistry^43,46^. U-series isotope measurements were conducted with a semi-static multi-cup setting according to Arps^48^ (HD), or^44^ (Mainz). Procedural blanks were frequently included in the extraction series and were determined to be < 0.4 fg for ^234^U and < 50 ag for ^230^Th. Ages were calculated using the half-lives of Cheng, et al.^49^. All ^230^Th/U-ages are reported relative to the year 1950 (BP). Uncertainties are reported at the 2σ-level, and do not include half-life errors.

### Major and trace element analysis

About 3 mg of each powdered carbonate were digested in 2 mL of 10% HNO_3_ for major and trace element analyses. Subsequently, concentrations of Ca, Mg, Sr, Ba, S, Fe and Mn of diluted aliquots were determined by ICP-OES at Heidelberg University. A procedural blank was prepared using the same protocol, and the elemental analysis revealed concentrations below the limit of quantification for all elements, with values < 1 µg L^−1^ for Mg, Sr, Ba, Fe, Mn, < 83 µg L^−1^ for S and < 10 µg L^−1^ for Ca. Quality control of the measurement was performed using reference materials SPS-SW1 and SPS-SW2 with recovery rates of ~ 100% for the analyzed elements. Quality control of the digestion of the carbonate material was performed with analyses of parallel aliquots of the limestone reference material ECRM 752-1. The recovery rate of the certified values was ~ 100% for the elements Ca, Mg, Sr, Fe and Mn, while a yield of ~ 90% was achieved for the element Ba and ~ 80% for the element Fe. The resulting element to Ca ratios are presented as molar ratios.

### Stable carbon and oxygen isotope measurements

For stable carbon and oxygen isotope analyses of carbonates, approximately 50–90 µg of powdered speleothem subsamples were analyzed using a ThermoFinnigan MAT253Plus gas source mass spectrometer equipped with a Thermo Fisher Scientific Kiel IV carbonate device at Heidelberg University (Institute of Earth Sciences). The δ^13^C and δ^18^O values are reported relative to VPDB through the analysis of an in-house standard (Solnhofen limestone, δ^13^C_VPDB_ =  + 1.38 ± 0.03‰ and δ^18^O_VPDB_ =  − 4.59 ± 0.06‰) calibrated to the reference material IAEA-603 (calcite; δ^13^C_VPDB_ =  + 2.46 ± 0.01‰ and δ^18^O_VPDB_ =  − 2.37 ± 0.04‰). External precisions (repeatable measurements of in-house standard) for δ^13^C and δ^18^O values were better than 0.03 and 0.06‰ (at 1*σ* level, n > 12), respectively.

## Supplementary Information

Supplementary Information.

## Data Availability

The raw data of the figures and tables presented in this paper are found in the Supplement and at the open data library PANGAEA (https://doi.pangaea.de/10.1594/PANGAEA.942956 and https://doi.pangaea.de/10.1594/PANGAEA.945784).
