# Supplementary material for: Mid- to late Holocene sea-level rise recorded in Hells Bells 234U/238U ratio and geochemical composition
Source: Sci Rep. 2023 Jun 20;13:10011. doi: 10.1038/s41598-023-36777-y (PMC10281970; doi:10.1038/s41598-023-36777-y)
Supplement: Supplementary file 1 — Supplementary Information. [file 41598_2023_36777_MOESM1_ESM.pdf]

Supplementary Information

"Mid- to late Holocene sea-level rise recorded in Hells Bells 234U/238U ratio and geochemical composition"

Nils Schorndorf, Norbert Frank, Simon M. Ritter, Sophie F. Warken, Christian Scholz, Frank Keppler, Denis Scholz, Michael Weber, Jeronimo Aviles Olguin, Wolfgang Stinnesbeck

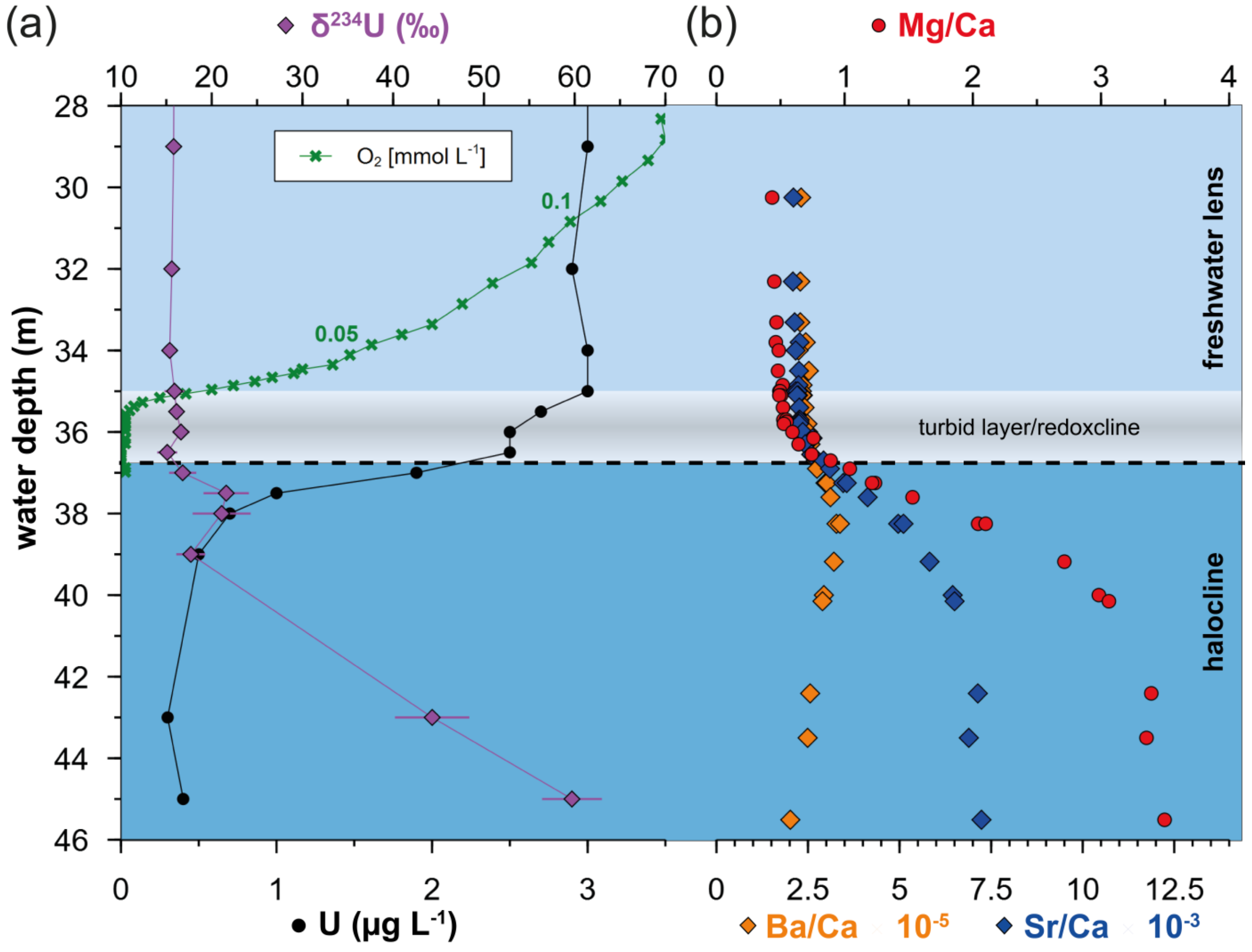

Supplementary Figure 1 – Geochemistry of recent water samples from El Zapote. U concentrations and $\delta^{234}U$ values (this study) are plotted next to Sr/Ca, Mg/Ca and Ba/Ca ratios as well as concentrations of dissolved oxygen taken from Ritter et al[1]. (a) Dissolved oxygen (green data points) decreases nearly linear from 30 m to concentrations below detection limit at ~35 m water depth, i.e. immediately above the turbid layer. $\delta^{234}U$ values increase from the top to the bottom of the halocline, while dissolved U shows a strong decrease across the redoxcline. (b) Sr/Ca, and

Mg/Ca ratios show increasing values from top to bottom of the halocline, whereas Ba/Ca ratios first present an increase, followed by a slight decline towards the bottom of the halocline. Uncertainties are given as 2σ margins. The dashed line separates the freshwater layer (above) from the halocline (below).

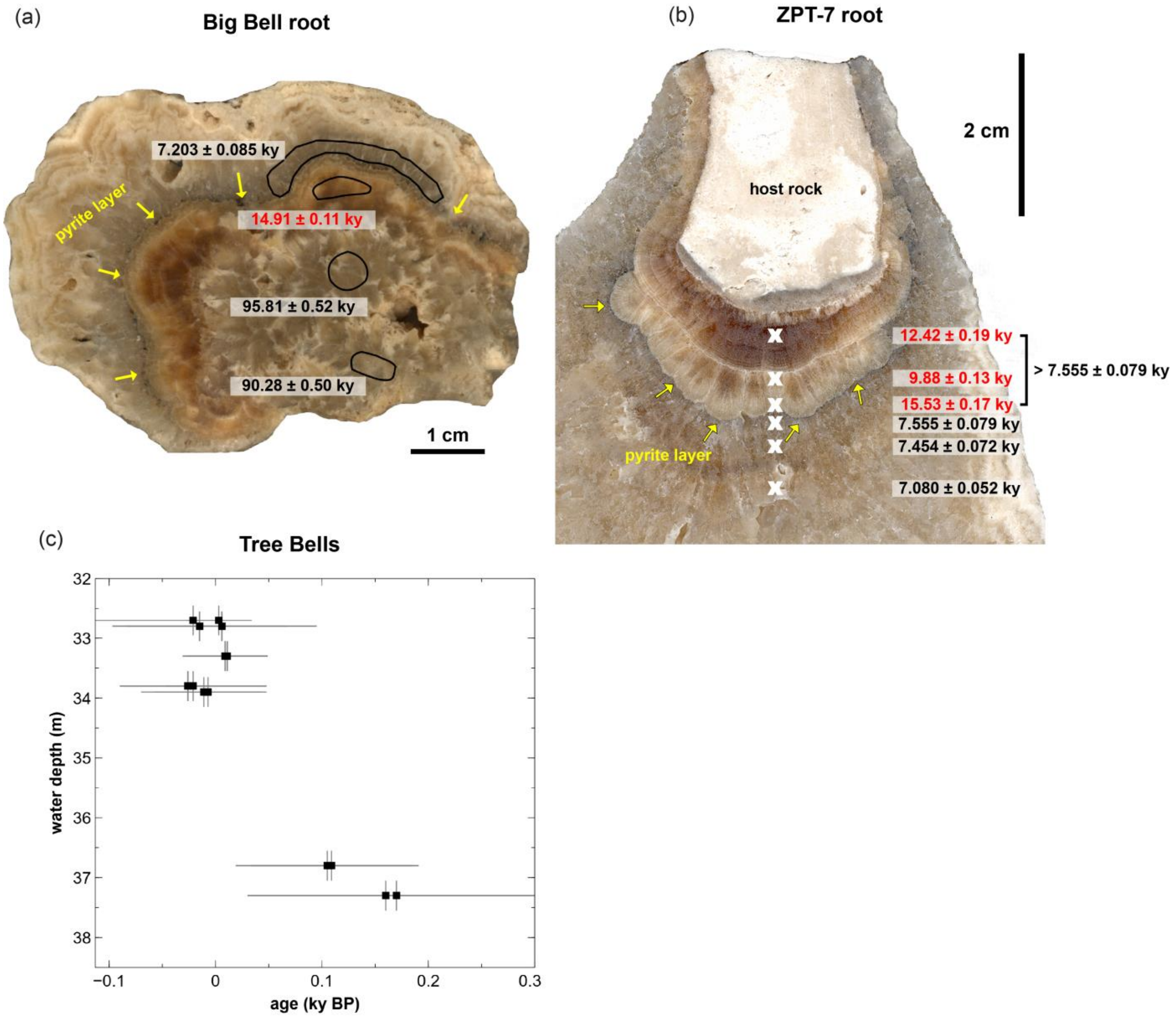

Supplementary Figure 2 – (a) ^{230}Th/U-dating results of Hells Bells speleothems from El Zapote cenote: Cross-section of the upper part of Big Bell showing sample location and corresponding ^{230}Th/U-ages. The arrows point to a thin black layer conformed by Fe-sulfide (pyrite). The photograph was taken from Ritter[2] and slightly modified. (b) Upper part of ZPT-7 showing the initial growth layers with corresponding ^{230}Th/U-ages (indicated by x). Note that a major age inversion is identified above and below a thin black Fe-sulfide (pyrite) layer, here indicated by arrows. (c) Age vs. water depth of Tree Bells. Age uncertainties are given as 2σ margins.

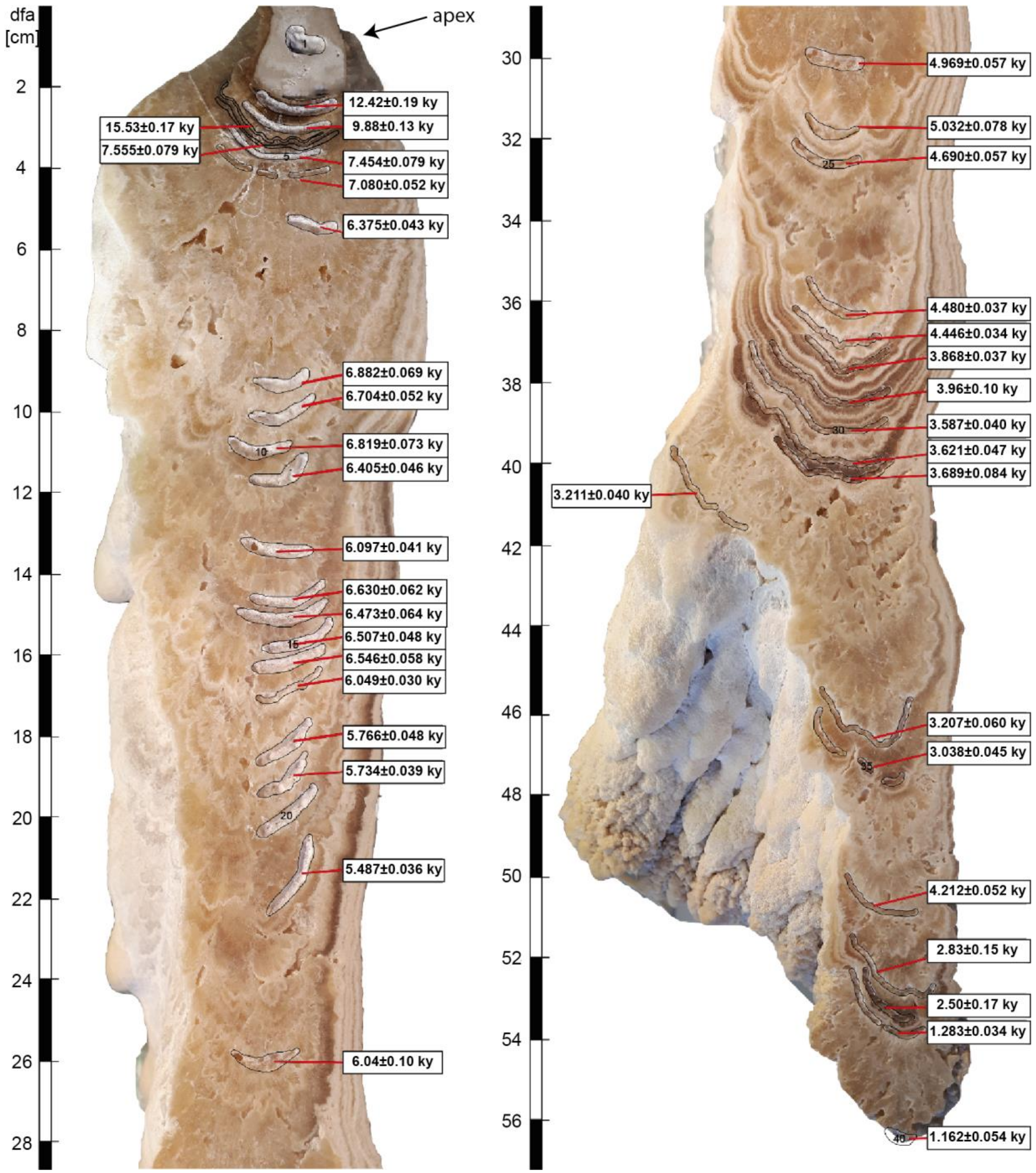

Supplementary Figure 3 – Sample spots and corresponding ^{230}Th/U-ages of Hells Bells specimen ZPT-7 that were vertically cut along their presumed growth axis. The results of ^{230}Th/U-dating can be found in the Supplementary Table 1. dfa = distance from the apex.

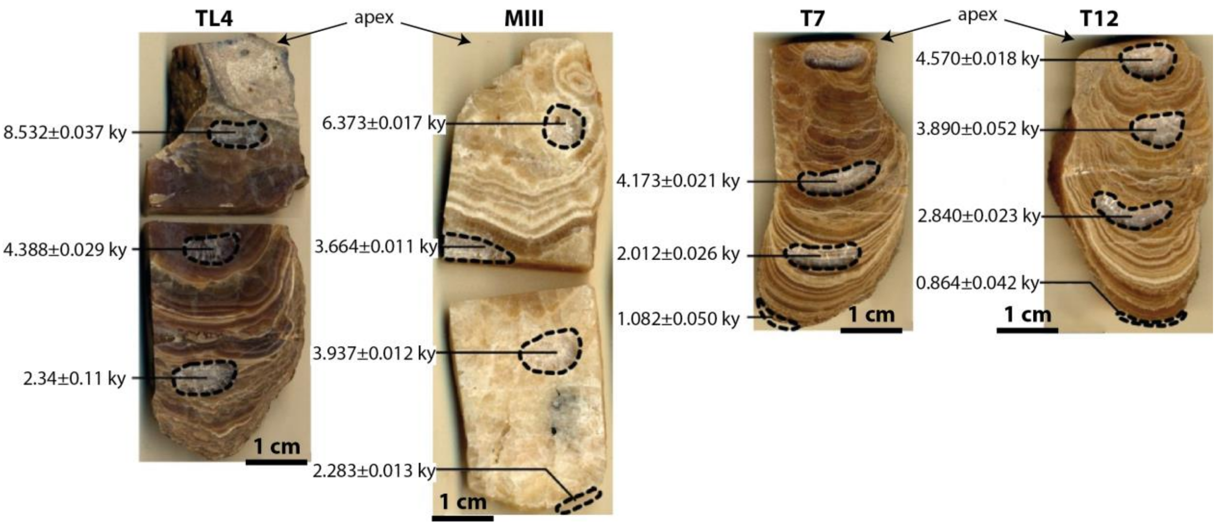

Supplementary Figure 4 – ^{230}Th/U-ages of the respective sampling spots of Hells Bells specimens from cenotes El Zapote (TL4), Maravilla (MIII) and Tortugas (T7 and T12) that were vertically cut along their presumed growth axis. The results of ^{230}Th/U-dating can be found in the Supplementary Table 1. The photographs of the Hells Bells slabs were taken from Ritter[2].

Supplementary Table 1 – $^{230}Th/U$ measurements of several Hells Bells specimens. Measurement and age uncertainties are 2σ analytical errors. cd1: Corrected $^{230}Th/U$-ages assume an initial ($^{230}Th/^{232}Th$) activity ratio of 2 ± 1. $^{230}Th/U$-ages are given relative to the year 1950 (BP). * activity ratio based on the half-lives of ^{230}Th and ^{234}U of Cheng et al[3], m : measured, 0 : initial, #: raw age before ($^{230}Th/^{232}Th$) activity correction model is applied.

Sample ID	Cenote	Label	^{238}U [ng/g]	^{232}Th [ng/g]	($^{230}Th/^{238}U$)*	($^{230}Th/^{232}Th$)*	$\delta^{234}U_m$ [‰]	Age [ky BP]#	Age^{cd1} [ky BP]	$\delta^{234}U_0$ [‰]	Distance from the apex [mm]	water depth [m]
Samples measured at Heidelberg University												
Big Bell 1 - 2	El Zapote	9201	1471.60±0.16	0.8309±0.0020	0.6016±0.0016	3274±12	25.2±1.8	95.85±0.52	95.81±0.52	33.0±2.4	-	-
Big Bell 1 - 1	El Zapote	9200	1576.94±0.14	1.9966±0.0043	0.5779±0.0018	1399.0±5.4	22.6±1.5	90.36±0.50	90.28±0.50	29.2±2.0	-	-
Big Bell 1 - 3	El Zapote	9202	843.670±0.088	1.7591±0.0032	0.13592±0.00060	200.7±1.0	48.5±3.2	15.056±0.088	14.91±0.11	50.6±3.3	-	-
Big Bell 1 - 4	El Zapote	9203	725.587±0.092	1.1971±0.0023	0.06852±0.00051	127.7±1.0	46.1±3.6	7.316±0.064	7.203±0.085	47.1±3.7	-	-
Big Bell 6 - 2	El Zapote	9222	537.935±0.045	3.4087±0.0067	0.03238±0.00036	15.71±0.18	25.0±2.3	3.432±0.040	2.99±0.23	25.2±2.4	~1800	-
Big Bell 6 - 1	El Zapote	9204	593.391±0.056	3.7008±0.0070	0.03276±0.00035	16.13±0.17	20.2±2.9	3.490±0.040	3.05±0.22	20.4±2.9	~1800	-
ZPT-7-2,7	El Zapote	9318	290.874±0.044	1.0479±0.0026	0.1152±0.0012	98.1±1.0	44.2±5.8	12.67±0.16	12.42±0.19	45.8±6.0	27	-
ZPT-7-3,2	El Zapote	9319	465.195±0.051	1.3422±0.0024	0.09265±0.00068	98.61±0.74	42.5±3.1	10.075±0.083	9.88±0.13	43.7±3.2	32	-
ZPT-7-3,5	El Zapote	9414	459.470±0.062	1.8434±0.0031	0.14373±0.00076	110.03±0.61	59.9±4.4	15.80±0.12	15.53±0.17	62.6±4.6	35	-
ZPT-7-3,7	El Zapote	9415	645.456±0.058	0.4708±0.0012	0.07114±0.00062	299.5±2.7	46.5±3.4	7.605±0.074	7.555±0.079	47.5±3.5	37	-
ZPT-7-3,8	El Zapote	9320	605.178±0.051	0.40214±0.00074	0.07061±0.00057	326.2±2.7	52.6±3.3	7.499±0.069	7.454±0.072	53.7±3.4	38	-
ZPT-7-4,3	El Zapote	9321	440.153±0.028	0.18939±0.00042	0.06649±0.00043	475.3±3.3	43.4±1.8	7.110±0.051	7.080±0.052	44.3±1.8	43	-
ZPT-7-5,6	El Zapote	9322	804.482±0.040	0.39749±0.00077	0.06004±0.00036	373.0±2.3	40.82±0.95	6.409±0.040	6.375±0.043	41.6±1.0	56	-
ZPT-7-9,4	El Zapote	9323	599.303±0.037	0.39619±0.00089	0.06469±0.00056	300.4±2.7	40.7±2.0	6.928±0.064	6.882±0.069	41.5±2.1	94	-
ZPT-7-10	El Zapote	9324	742.564±0.047	0.3751±0.0013	0.06292±0.00043	384.7±3.0	39.5±1.4	6.738±0.050	6.704±0.052	40.3±1.4	100	-
ZPT-7-11	El Zapote	9325	680.951±0.062	0.5187±0.0021	0.06399±0.00059	258.4±2.6	37.6±2.3	6.871±0.068	6.819±0.073	38.3±2.3	110	-
ZPT-7-11,5	El Zapote	9326	780.324±0.047	0.3262±0.0011	0.06016±0.00039	443.3±3.2	39.0±1.2	6.434±0.043	6.405±0.046	39.7±1.2	115	-
ZPT-7-13,5	El Zapote	9327	634.679±0.036	0.16242±0.00040	0.05704±0.00034	683.5±4.4	34.5±1.3	6.115±0.039	6.097±0.041	35.1±1.3	135	-
ZPT-7-14,7	El Zapote	9328	664.897±0.038	0.8379±0.0014	0.06261±0.00038	152.7±1.0	37.6±1.1	6.717±0.043	6.630±0.062	38.4±1.1	147	-
ZPT-7-15,2	El Zapote	9329	933.490±0.084	0.4252±0.0014	0.06056±0.00054	409.0±3.9	35.1±1.7	6.504±0.061	6.473±0.064	35.7±1.7	152	-

Sample ID	Cenote	Label	^{238}U [ng/g]	^{232}Th [ng/g]	$(^{230}Th/^{238}U)$*	$(^{230}Th/^{232}Th)$*	$\delta^{234}U_m$ [‰]	Age [ky BP]#	Age^{cd1} [ky BP]	$\delta^{234}U_0$ [‰]	Distance from the apex [mm]	water depth [m]
ZPT-7-115,8	El Zapote	9330	535.00±0.022	0.24119±0.00045	0.06081±0.00040	414.1±2.8	34.2±1.1	6.538±0.046	6.507±0.048	34.8±1.1	158	-
ZPT-7-16,3	El Zapote	9331	470.396±0.024	0.27141±0.00058	0.06129±0.00048	325.6±2.7	35.1±1.1	6.586±0.054	6.546±0.058	35.7±1.1	163	-
ZPT-7-16,9	El Zapote	9332	1248.122±0.066	0.35221±0.00063	0.05671±0.00025	616.2±2.9	35.99±0.74	6.068±0.028	6.049±0.030	36.62±0.75	169	-
ZPT-7-18,2	El Zapote	9333	443.348±0.018	0.27632±0.00057	0.05407±0.00038	268.0±2.0	30.2±1.2	5.809±0.043	5.766±0.048	30.7±1.3	182	-
ZPT-7-19,1	El Zapote	9334	628.348±0.034	0.24233±0.00044	0.05355±0.00032	425.3±2.6	28.6±1.1	5.761±0.036	5.734±0.039	29.1±1.1	191	-
ZPT-7-21,5	El Zapote	9336	1055.126±0.058	0.30942±0.00064	0.05131±0.00031	540.2±3.5	29.2±1.0	5.507±0.035	5.487±0.036	29.6±1.0	215	-
ZPT-7-26	El Zapote	9337	644.173±0.093	0.3715±0.0015	0.05670±0.00086	301.0±4.7	34.2±4.0	6.079±0.098	6.04±0.10	34.8±4.0	260	-
ZPT-7-30,1	El Zapote	9338	519.821±0.047	0.4303±0.0010	0.04692±0.00042	174.1±1.6	27.6±2.6	5.027±0.049	4.969±0.057	28.0±2.7	301	-
ZPT-7-31,8	El Zapote	9339	383.489±0.025	0.31460±0.00083	0.04756±0.00065	178.5±2.5	29.4±2.5	5.089±0.073	5.032±0.078	29.8±2.5	318	-
ZPT-7-32,5	El Zapote	9340	735.215±0.069	0.4417±0.0014	0.04416±0.00046	225.4±2.5	25.4±2.3	4.732±0.052	4.690±0.057	25.8±2.4	325	-
ZPT-7-36,2	El Zapote	9341	749.749±0.047	0.3941±0.0010	0.04215±0.00029	246.5±1.8	23.5±1.2	4.517±0.032	4.480±0.037	23.8±1.2	362	-
ZPT-7-36,8	El Zapote	9342	917.724±0.049	0.34974±0.00069	0.04171±0.00027	336.9±2.3	22.7±1.3	4.472±0.031	4.446±0.034	23.0±1.3	368	-
ZPT-7-37,5	El Zapote	9343	1139.734±0.098	0.4232±0.0012	0.03640±0.00030	302.0±2.6	20.1±1.4	3.894±0.034	3.868±0.037	20.4±1.4	375	-
ZPT-7-38,3	El Zapote	9344	209.893±0.016	0.41887±0.00089	0.03826±0.00069	59.0±1.1	21.4±2.7	4.095±0.078	3.96±0.10	21.6±2.7	383	-
ZPT-7-38,9	El Zapote	9345	1027.576±0.079	0.4133±0.0011	0.03397±0.00034	259.7±2.7	22.8±1.3	3.615±0.037	3.587±0.040	23.0±1.3	389	-
ZPT-7-39,9	El Zapote	9346	1116.020±0.060	1.2302±0.0029	0.03451±0.00024	95.67±0.70	16.31±0.69	3.698±0.027	3.621±0.047	16.48±0.70	399	-
ZPT-7-40,3	El Zapote	9347	709.794±0.042	1.5744±0.0034	0.03594±0.00027	49.82±0.39	19.8±1.2	3.844±0.031	3.689±0.084	20.0±1.2	403	-
ZPT-7-41,4	El Zapote	9348	919.159±0.049	0.7779±0.0020	0.03076±0.00024	111.82±0.91	20.0±1.2	3.271±0.027	3.211±0.040	20.2±1.3	414	-
ZPT-7-46,4	El Zapote	9349	602.789±0.058	0.8027±0.0018	0.03108±0.00034	71.69±0.81	21.9±1.8	3.300±0.038	3.207±0.060	22.2±1.8	464	-
ZPT-7-47,5	El Zapote	9350	892.014±0.078	0.8398±0.0020	0.02926±0.00028	95.60±0.93	20.3±2.2	3.104±0.031	3.038±0.045	20.5±2.2	475	-
ZPT-7-51	El Zapote	9351	653.025±0.042	0.6291±0.0013	0.03987±0.00034	127.4±1.1	19.9±1.9	4.280±0.039	4.212±0.052	20.1±1.9	510	-
ZPT-7-52,6	El Zapote	9352	622.131±0.037	2.5018±0.0038	0.02930±0.00030	22.43±0.24	19.6±1.8	3.111±0.034	2.83±0.15	19.8±1.8	526	-
ZPT-7-53,4	El Zapote	9353	546.408±0.034	2.4705±0.0059	0.02654±0.00035	18.07±0.24	16.7±2.1	2.817±0.039	2.50±0.17	16.8±2.1	534	-
ZPT-7-53,9	El Zapote	9354	820.952±0.074	0.5667±0.0018	0.01298±0.00021	57.6±1.0	18.0±2.6	1.332±0.023	1.283±0.034	18.1±2.6	539	-
ZPT-7-56,7	El Zapote	9355	1156.683±0.095	1.4461±0.0027	0.01220±0.00029	29.95±0.70	15.6±1.6	1.250±0.031	1.162±0.054	15.7±1.7	567	-
Tree Bell-1	El Zapote	9290	694.98±0.51	0.5705±0.0025	0.00098±0.00011	3.70±0.40	17.5±2.3	0.037±0.011	−0.021±0.031	17.5±2.3	-	31.3

Sample ID	Cenote	Label	^{238}U [ng/g]	^{232}Th [ng/g]	$(^{230}Th/^{238}U)$*	$(^{230}Th/^{232}Th)$*	$\delta^{234}U_m$ [‰]	Age [ky BP]#	Age^{cd1} [ky BP]	$\delta^{234}U_0$ [‰]	Distance from the apex [mm]	water depth [m]
Tree Bell-2	El Zapote	9291	695.27±0.11	0.5533±0.0023	0.00119±0.00013	4.66±0.50	18.3±2.3	0.059±0.014	0.003±0.031	18.3±2.3	-	31.3
Tree Bell-3	El Zapote	9288	1012.37±0.12	2.448±0.012	0.00208±0.00010	2.65±0.13	18.1±1.4	0.155±0.011	−0.015±0.082	18.1±1.4	-	32.8
Tree Bell-4	El Zapote	9289	1112.37±0.12	2.841±0.012	0.00236±0.00018	2.85±0.21	18.3±2.4	0.186±0.019	0.006±0.089	18.3±2.4	-	32.8
Tree Bell-5	El Zapote	9286	733.189±0.090	0.8148±0.0027	0.001446±0.000088	4.01±0.24	15.4±1.3	0.0874±0.0094	0.009±0.040	15.4±1.3	-	33.3
Tree Bell-6	El Zapote	9287	811.121±0.081	0.8503±0.0024	0.001421±0.000094	4.19±0.28	17.6±1.4	0.084±0.010	0.011±0.038	17.6±1.4	-	33.3
Tree Bell-7	El Zapote	9278	887.973±0.068	0.4894±0.0015	0.000750±0.000065	4.20±0.36	19.4±1.3	0.0123±0.0069	−0.026±0.021	19.4±1.3	-	33.8
Tree Bell-8	El Zapote	9279	776.909±0.059	1.5608±0.0043	0.001752±0.000078	2.69±0.12	14.0±1.3	0.1206±0.0085	−0.021±0.069	14.0±1.3	-	33.8
Tree Bell-9	El Zapote	9284	761.028±0.080	1.1059±0.0040	0.001517±0.000071	3.22±0.15	16.9±1.7	0.0947±0.0075	−0.007±0.050	16.9±1.7	-	33.9
Tree Bell-10	El Zapote	9285	847.94±0.15	1.4545±0.0063	0.00165±0.00015	2.96±0.27	15.6±2.3	0.109±0.016	−0.011±0.059	15.6±2.3	-	33.9
Tree Bell-11	El Zapote	9282	1447.25±0.11	3.580±0.013	0.003226±0.000092	4.03±0.12	16.6±1.0	0.279±0.010	0.105±0.086	16.6±1.0	-	36.8
Tree Bell-12	El Zapote	9283	1520.63±0.13	3.297±0.013	0.003064±0.000094	4.36±0.13	15.1±1.2	0.262±0.010	0.109±0.076	15.1±1.2	-	36.8
Tree Bell-13	El Zapote	9280	1429.94±0.14	5.110±0.014	0.00459±0.00015	3.95±0.13	15.7±1.2	0.425±0.017	0.17±0.13	15.7±1.2	-	37.3
Tree Bell-14	El Zapote	9281	1494.38±0.15	5.795±0.015	0.00468±0.00014	3.71±0.11	15.3±1.5	0.436±0.015	0.16±0.14	15.3±1.5	-	37.3
ZPT-MUDE-05-T	El Zapote	11720	1724.576±0.094	0.8743±0.0014	0.012576±0.000066	76.14±0.42	18.37±0.33	1.3549±0.0071	1.319±0.019	18.44±0.33	-	-
ZPT-MUDE-05-R	El Zapote	11721	425.361±0.023	0.28772±0.00061	0.03409±0.00026	153.4±1.2	23.2±1.1	3.694±0.029	3.647±0.038	23.5±1.1	-	-
ZPT-MUDE-04	El Zapote	11722	391.677±0.018	2.8199±0.0050	0.06120±0.00027	25.89±0.12	30.88±0.73	6.671±0.030	6.17±0.25	31.43±0.74	-	-
ZPT-MUDE-03-T	El Zapote	11723	296.223±0.018	1.7898±0.0043	0.05999±0.00040	30.24±0.21	31.5±1.7	6.532±0.047	6.11±0.22	32.1±1.7	-	-
ZPT-MUDE-02-T	El Zapote	11724	1305.827±0.050	0.8291±0.0013	0.009100±0.000083	43.67±0.41	17.91±0.47	0.9793±0.0090	0.935±0.024	17.95±0.47	-	-
ZPT-MUDE-01-T-OC	El Zapote	11725	1021.805±0.047	0.5793±0.0011	0.008813±0.000054	47.41±0.31	17.84±0.53	0.94830.0058	0.909±0.021	17.89±0.53	-	-
ZPT-MUDE-03-T	El Zapote	11726	850.562±0.040	0.6496±0.0013	0.01271±0.00010	50.90±0.42	16.50±0.46	1.372±0.011	1.318±0.029	16.56±0.46	-	-
Samples measured at Mainz University												
TL4-1	El Zapote	-	670.7±4.2	0.5320±0.0034	0.07826±0.00032	303.39±0.83	31.4±0.5	8.587±0.025	8.532±0.037	31.5±0.5	16	35.8
TL4-3	El Zapote	-	470.2±2.9	0.2610±0.0018	0.04109±0.00026	228.2±1.2	25.9±0.4	4.427±0.021	4.388±0.029	25.9±0.4	36	35.8

Sample ID	Cenote	Label	^{238}U [ng/g]	^{232}Th [ng/g]	$(^{230}Th/^{238}U)$*	$(^{230}Th/^{232}Th)$*	$\delta^{234}U_m$ [‰]	Age [ky BP]#	Age^{cd1} [ky BP]	$\delta^{234}U_0$ [‰]	Distance from the apex [mm]	water depth [m]
TL4-2	El Zapote	-	946.5±5.8	3.117±0.020	0.0223±0.0011	22.685±0.078	21.5±0.3	2.5718±0.0085	2.34±0.11	21.5±0.3	58	35.8
MIII-1	Maravilla	-	1508.2±9.3	0.3153±0.0020	0.05975±0.00015	875.6±2.0	41.3±0.4	6.387±0.016	6.373±0.017	41.3±0.4	14	29.4
MIII-2	Maravilla	-	1826.2±11.5	0.2783±0.0018	0.03437±0.00010	691.3±1.7	21.4±0.4	3.6741±0.0093	3.664±0.011	21.4±0.4	45	29.4
MIII-3	Maravilla	-	1715.3±10.4	0.2986±0.0019	0.03682±0.00011	648.5±1.6	20.6±0.3	3.949±0.010	3.937±.0.012	20.6±0.3	35	29.4
MIII-4	Maravilla	-	3097.6±19.5	0.9093±0.0060	0.02179±0.00011	228.88±0.69	21.1±0.3	2.3038±0.0068	2.283±0.013	21.1±0.3	70	29.4
T7-2	Tortugas	-	1009.2±6.3	0.4350±0.0029	0.03934±0.00019	280.8±1.1	30.8±0.4	4.203±0.015	4.173±0.021	30.8±0.4	23	32.9
T7-3	Tortugas	-	1145.5±7.1	0.8185±0.0053	0.01940±0.00024	84.95±0.30	26.2±0.3	2.0619±0.0072	2.012±0.026	26.2±0.3	36	32.9
T7-4	Tortugas	-	1312.3±8.8	1.816±0.013	0.01074±0.00046	25.68±0.20	22.8±1.9	1.1782±0.0098	1.082±0.050	22.8±1.9	46	32.9
T12-1	Tortugas	-	925.3±5.7	0.3373±0.0021	0.04296±0.00016	362.14±0.93	31.3±0.4	4.595±0.013	4.570±0.018	31.3±0.4	4	30.5
T12-2	Tortugas	-	704.3±4.3	0.9820±0.0061	0.03663±0.00046	82.23±0.27	27.1±0.7	3.987±0.014	3.890±0.052	27.1±0.7	16	30.5
T12-3	Tortugas	-	851.0±5.4	0.4882±0.0032	0.02701±0.00021	145.85±0.50	25.8±0.5	2.880±0.011	2.840±0.023	25.8±0.5	30	30.5
T12-4	Tortugas	-	899.2±5.6	1.0715±0.0070	0.00873±0.00039	24.38±0.14	24.4±0.5	0.9475±0.0058	0.864±0.042	24.4±0.5	48	30.5

Supplementary Table 2 – Geochemical data of Hells Bells samples from different cenotes taken along their presumed growth axis. Uncertainties of stable isotope analyses $\delta^{13}C$ and $\delta^{18}O$ are <0.03‰ and <0.06‰, respectively. *Data recently published by Ritter et al[1].

Sample ID	Cenote	water depth [m]	Distance from the apex [mm]	Mg/Ca [10^{-2}]	Sr/Ca [10^{-4}]	Ba/Ca [10^{-5}]	Fe/Ca [10^{-5}]	Mn/Ca [10^{-5}]	S/Ca [10^{-3}]	$\delta^{13}C$ [‰VPDB]	$\delta^{18}O$ [‰VPDB]
ZPT-7-host rock	El Zapote	-	10	3.30	1.9	0.17	5.1	2.1	0.4	−4.85	−3.75
ZPT-7-2,7	El Zapote	-	27	5.09	14.1	4.4	4.1	34.1	-	−7.62	−4.34
ZPT-7-3,2	El Zapote	-	32	5.09	13.8	4.0	4.4	25.8	0.1	−8.93	−4.88
ZPT-7-3,8	El Zapote	-	38	3.61	9.2	2.82	7.1	7.6	1.7	−13.22	−5.70
ZPT-7-4,3	El Zapote	-	43	3.99	12.5	4.66	3.5	4.6	0.5	−13.10	−5.22
ZPT-7-5,6	El Zapote	-	56	2.94	9.5	3.56	5.6	1.0	1.1	−12.60	−5.57
ZPT-7-9,4	El Zapote	-	94	2.69	9.2	3.47	4.2	2.4	1.3	−12.83	−5.70
ZPT-7-10	El Zapote	-	100	2.79	8.7	3.25	3.4	2.1	1.2	−12.74	−5.81
ZPT-7-11	El Zapote	-	110	3.10	9.4	3.56	6.3	7.3	1.3	−12.94	−5.74
ZPT-7-11,5	El Zapote	-	115	2.94	7.9	2.68	2.4	1.3	1.1	−12.89	−5.83
ZPT-7-13,5	El Zapote	-	135	3.17	8.9	3.06	1.7	2.3	1.0	−12.86	−5.50
ZPT-7-14,7	El Zapote	-	147	2.81	7.3	2.40	11.2	4.3	1.2	−12.79	−5.79
ZPT-7-15,2	El Zapote	-	152	2.38	7.4	2.77	1.6	1.1	1.2	−12.94	−5.71
ZPT-7-15,8	El Zapote	-	158	2.90	8.2	2.91	2.6	1.6	1.1	−12.86	−5.60
ZPT-7-16,3	El Zapote	-	163	3.07	8.2	2.87	2.9	1.6	1.1	−12.91	−5.57
ZPT-7-16,9	El Zapote	-	169	2.65	7.1	2.63	4.0	1.1	1.4	−13.04	−5.32
ZPT-7-18,2	El Zapote	-	182	3.16	6.6	2.20	2.2	2.1	1.4	−13.20	−5.52
ZPT-7-19,1	El Zapote	-	191	3.35	6.3	2.20	2.2	2.3	1.4	−13.21	−5.28
ZPT-7-20	El Zapote	-	200	2.90	5.5	2.06	6.0	1.2	1.5	−13.26	−5.28
ZPT-7-21,5	El Zapote	-	215	3.19	6.6	2.65	0.8	1.0	1.9	−13.30	−5.32
ZPT-7-26	El Zapote	-	260	3.48	6.6	2.22	12.3	2.7	1.9	−13.59	−5.14
ZPT-7-30,1	El Zapote	-	301	3.60	7.3	2.64	2.1	1.5	1.9	−13.67	−4.84
ZPT-7-31,8	El Zapote	-	318	4.0	7.6	2.83	2.0	1.8	2.7	−13.67	−4.65
ZPT-7-32,5	El Zapote	-	325	3.04	7.6	3.13	0.9	0.7	2.9	−13.50	−5.23
ZPT-7-36,2	El Zapote	-	362	3.44	7.1	2.67	0.5	0.8	2.4	−13.63	−5.04
ZPT-7-36,8	El Zapote	-	368	2.94	6.5	2.24	11.3	1.2	2.2	−13.34	−4.99
ZPT-7-37,5	El Zapote	-	375	3.05	5.1	1.80	5.6	4.5	2.6	−13.58	−4.99
ZPT-7-38,3	El Zapote	-	383	3.97	6.4	2.03	0.7	3.9	2.4	−14.32	−5.05
ZPT-7-38,9	El Zapote	-	389	2.87	5.7	2.20	0.5	1.1	2.8	−13.62	−4.94
ZPT-7-39,9	El Zapote	-	399	2.91	5.7	2.26	4.4	5.5	2.8	−13.65	−5.02
ZPT-7-40,3	El Zapote	-	403	3.44	6.0	2.22	3.7	7.2	2.7	−13.97	−4.78
ZPT-7-41,4	El Zapote	-	414	3.17	5.2	1.67	2.0	1.8	2.2	−13.38	−4.86
ZPT-7-46,4	El Zapote	-	464	3.16	5.7	2.04	2.3	1.6	2.7	−13.42	−4.85
ZPT-7-47,5	El Zapote	-	475	3.14	5.0	1.66	2.0	2.3	2.8	−13.44	−4.80
ZPT-7-51	El Zapote	-	510	2.93	5.6	1.94	6.3	1.3	2.8	−13.37	−5.09
ZPT-7-52,6	El Zapote	-	526	3.28	5.9	1.95	4.4	3.6	2.7	−13.80	−4.99
ZPT-7-53,4	El Zapote	-	534	3.34	5.4	1.76	7.6	9.2	2.9	−14.01	−4.96
ZPT-7-53,9	El Zapote	-	539	2.88	3.9	1.00	7.1	3.8	2.0	−13.28	−4.95

Sample ID	Cenote	water depth	Distance from the apex	Mg/Ca	Sr/Ca	Ba/Ca	Fe/Ca	Mn/Ca	S/Ca	$\delta^{13}C$	$\delta^{18}O$
		[m]	[mm]	$[10^{-2}]$	$[10^{-4}]$	$[10^{-5}]$	$[10^{-5}]$	$[10^{-5}]$	$[10^{-3}]$	[‰VPDB]	[‰VPDB]
ZPT-7-56,7	El Zapote	-	567	2.58	3.9	1.16	6.0	3.9	2.6	−13.58	−5.04
Big Bell 1 - 1	El Zapote	-	-	2.57	7.6	1.95	46.4	2.5	1.8	−11.99	−6.39
Big Bell 1 - 2	El Zapote	-	-	2.83	7.6	2.05	22.6	1.4	1.6	−12.03	−6.10
Big Bell 1 - 3	El Zapote	-	-	6.10	14.1	3.79	5.1	23.8	-	−8.15	−4.19
Big Bell 1 - 4	El Zapote	-	-	4.80	12.7	4.10	11.3	2.4	-	−12.67	−5.40
Big Bell 6 - 1	El Zapote	-	~1800	3.58	6.9	2.15	3.9	10.9	3.4	-	-
Big Bell 6 - 2	El Zapote	-	~1800	3.46	6.5	2.03	4.2	10.3	3.1	−14.27	−4.90
Tree Bell-2	El Zapote	31.3	-	2.14*	4.16*	1.27*	4.3*	2.1*	2.78*	−13.47*	−4.34
Tree Bell-3	El Zapote	32.8	-	2.56*	4.10*	1.19*	5.2*	2.6*	2.69*	−13.69*	−4.83
Tree Bell-5	El Zapote	33.3	-	2.20*	4.27*	1.30*	3.8*	2.4*	3.05*	−13.82*	−4.92
Tree Bell-6	El Zapote	33.3	-	2.23*	3.85*	1.14*	3.0*	1.6*	2.54*	−13.43*	−4.92
Tree Bell-8	El Zapote	33.8	-	2.08*	3.79*	1.12*	3.5*	2.4*	2.72*	−13.52*	−4.96
Tree Bell-9	El Zapote	33.9	-	2.32*	4.00*	1.18*	3.9*	2.2*	2.76*	−13.68*	−4.84
Tree Bell-11	El Zapote	36.8	-	2.23*	3.40*	0.88*	11.3*	3.9*	3.14*	−12.87*	−5.40
Tree Bell-12	El Zapote	36.8	-	2.11*	3.41*	0.88*	10.6*	2.8*	3.02*	−12.85*	−5.35
Tree Bell-13	El Zapote	37.3	-	2.40*	3.73*	0.92*	6.2*	3.2*	3.17*	−12.99*	−5.13
TL4-1	El Zapote	35.8	16	3.41	6.7	2.07	4.1	3	2	−13.35	−5.43
TL4-3	El Zapote	35.8	36	3.18	6.8	2.13	1.8	2.1	3	−14.02	−4.88
TL4-2	El Zapote	35.8	58	2.4	4.4	1.31	9	8.1	3.3	−13.36	−5.45
MIII-1	Maravilla	29.4	14	3.15	8.2	2.64	1.7	1.9	3.8	−12.99	−5.77
MIII-2	Maravilla	29.4	45	2.9	3.7	0.71	2.3	5.8	2.8	−12.75	−5.19
MIII-3	Maravilla	29.4	35	3.08	4.3	0.98	1.9	3.2	2.8	−12.87	−5.15
MIII-4	Maravilla	29.4	70	2.89	4.2	0.98	0.8	2.1	2.5	−12.68	−5.09
T7-2	Tortugas	32.9	23	3.3	5.3	1.26	6.6	1.7	2	−11.37	−5.21
T7-3	Tortugas	32.9	36	3.01	4.4	1.02	9.1	4	2	−11.55	−5.21
T7-4	Tortugas	32.9	46	2.17	3.6	0.94	5.4	1.8	2.3	−12.43	−5.69
T12-1	Tortugas	30.5	4	3.26	5.6	1.34	4.7	1.1	2.1	−11.07	−5.13
T12-2	Tortugas	30.5	16	3.89	5.6	1.39	4	1.5	2.4	−11.24	−5.09
T12-3	Tortugas	30.5	30	3.14	4.4	1.1	10.5	2.5	2.3	−11.37	−5.23
T12-4	Tortugas	30.5	48	2.62	3.9	0.95	5	1.8	2.5	−11.56	−5.21

References

1 Ritter, S. M. *et al.* Subaqueous speleothems (Hells Bells) formed by the interplay of pelagic redoxcline biogeochemistry and specific hydraulic conditions in the El Zapote sinkhole, Yucatan Peninsula, Mexico. *Biogeosciences* **16**, 2285-2305, doi:10.5194/bg-16-2285-2019 (2019).

2 Ritter, S. M. *Unravelling the formation of Hells Bells: underwater speleothems from the Yucatán Peninsula in Mexico* Ph.D. thesis, Heidelberg University, (2020).

3 Cheng, H. *et al.* Improvements in 230Th dating, 230Th and 234U half-life values, and U–Th isotopic measurements by multi-collector inductively coupled plasma mass spectrometry. *Earth Planet. Sci. Lett.* **371-372**, 82-91, doi:10.1016/j.epsl.2013.04.006 (2013).
